# Amyloid, Crohn’s disease, and Alzheimer’s disease - are they linked?

**DOI:** 10.3389/fcimb.2024.1393809

**Published:** 2024-05-08

**Authors:** Anna Duda-Madej, Jakub Stecko, Natalia Szymańska, Agnieszka Miętkiewicz, Marta Szandruk-Bender

**Affiliations:** ^1^ Department of Microbiology, Faculty of Medicine, Wroclaw Medical University, Wrocław, Poland; ^2^ Faculty of Medicine, Wroclaw Medical University, Wrocław, Poland; ^3^ Department of Pharmacology, Faculty of Medicine, Wroclaw Medical University, Wrocław, Poland

**Keywords:** inflammatory bowel disease, Crohn’s disease, neurodegenerative disease, Alzheimer’s disease, β-amyloid (Aβ), curli, microbiota, gut-microbial-brain axis

## Abstract

Crohn’s disease (CD) is a chronic inflammatory disease that most frequently affects part of the distal ileum, but it may affect any part of the gastrointestinal tract. CD may also be related to systemic inflammation and extraintestinal manifestations. Alzheimer’s disease (AD) is the most common neurodegenerative disease, gradually worsening behavioral and cognitive functions. Despite the meaningful progress, both diseases are still incurable and have a not fully explained, heterogeneous pathomechanism that includes immunological, microbiological, genetic, and environmental factors. Recently, emerging evidence indicates that chronic inflammatory condition corresponds to an increased risk of neurodegenerative diseases, and intestinal inflammation, including CD, increases the risk of AD. Even though it is now known that CD increases the risk of AD, the exact pathways connecting these two seemingly unrelated diseases remain still unclear. One of the key postulates is the gut-brain axis. There is increasing evidence that the gut microbiota with its proteins, DNA, and metabolites influence several processes related to the etiology of AD, including β-amyloid abnormality, Tau phosphorylation, and neuroinflammation. Considering the role of microbiota in both CD and AD pathology, in this review, we want to shed light on bacterial amyloids and their potential to influence cerebral amyloid aggregation and neuroinflammation and provide an overview of the current literature on amyloids as a potential linker between AD and CD.

## 1 Crohn’s disease

### 1.1 Crohn’s disease – general information

Inflammatory bowel disease (IBD) includes ulcerative colitis (UC) and Crohn’s disease (CD). The two disease differ in their symptoms, radiographic appearance and histological changes. In this review, we focused on CD, as the histological changes in its course involve the entire thickness of the intestinal wall, characterized by localized lymphocytic infiltration, granulomas and fibrosis. In contrast to UC, in which lesions are limited to superficial inflammation with the presence of crypt abscesses (Le Berre et al., 2020).

CD is characterized by chronic, transmural, and mostly granulomatous inflammation of the gastrointestinal tract. CD usually affects the distal ileum, cecum, or colon but can affect any part of the gastrointestinal tract. Typically, the CD has an intermittent course with periods of acute flares and remissions. Clinical symptoms vary depending on the severity and section of the gastrointestinal tract involved, ranging from mild to severe. The main symptoms are abdominal pain, diarrhea, low-grade fever, fatigue, unintended weight loss, and malnutrition. Rectal bleeding during CD is less common but can occur when the distal colon is involved. As the disease progresses, the chronic inflammatory process of the intestines disturbs their function, intestinal complications appear, and then – also extraintestinal symptoms (Guan, 2019; Petagna et al., 2020).

Despite comprehensive studies, the exact cause of CD is still not fully understood. Current consensus considers a multifactorial and heterogeneous pathogenesis of CD. It is believed that a complex interaction between genetic, environmental, and microbial factors may lead to dysregulated and enhanced immune response (Sobieszczańska et al., 2019; Ranasinghe; and Hsu, 2023).

### 1.2 Immunological factors in the pathogenesis of Crohn’s disease

An unrestrainable immune response against luminal antigens, leading to tissue inflammation, is an indisputable factor in the pathogenesis of CD. During chronic inflammation, immune cells, including CD4^+^ and CD8^+^ T helper (Th) cells, infiltrate and accumulate in the gastrointestinal tract of CD patients. Therefore, dysregulation of various components of the immune system is invariably found in the mucosa of CD patients (Petagna et al., 2020). One of the most expressed alterations that mediate abnormal immune response and subsequent inflammation in the intestinal mucosa include increasing migration, proliferation, and activation of Th cells, especially Th1 and Th17 cells. As a result, there is an upregulation of the synthesis and release of various proinflammatory mediators. Numerous studies have shown increased amounts of mRNA for TNF-α, IL-2, IL-6, IL-8, IL-12, IL-17, IL-21, IL-22, IL-23, CCL20, and chemerin, and increased concentrations of these markers in serum and intestinal mucosa biopsies from CD patients (Guan, 2019).

The role of Th17 cell subpopulation in the pathogenesis of CD is increasingly emphasized. Th17 cells are controlled by Treg cells, which inhibit the former’s excessive immune response, they must remain in dynamic balance. When it is lost and shifts towards the proinflammatory Th17 cells, which constantly accumulate, proinflammatory cytokines are continuously synthesized and released. This exceeds the immune tolerance of Treg and leads to persistent mucosal inflammation (Szandruk-Bender et al., 2022a; Chen et al., 2023; Szandruk-Bender et al., 2023). Intestinal Tregs have T-cell receptors (TCRs) specific for intestinal antigens. They are essential for suppressing the immune response against the gut microbiota (Choi et al., 2022). In addition, Treg stimulate the development of intestinal stem cells (ISCs), ensuring the integrity of the intestinal epithelium and maintaining intestinal homeostasis (Harada et al., 2022). Treg can enter the central nervous system via three routes, through: (i) the blood-brain barrier (BBB) (into the perivascular space); (ii) the subarachnoid space in the meninges, and (iii) the choroid plexus (into the cerebrospinal fluid). Treg accumulating in damaged areas infiltrate the brain and, being able to interact with microglia, exacerbate inflammation within the nervous system contributing to the development of neurodegenerative diseases (Ma et al., 2024).

Both Th17 and Treg cells are differentiated from naive CD4+ T cells under the influence of relevant transcription factors and microenvironmental cues. Differentiation of Th17 cells is driven by retinoic acid related orphan receptor γt (RORγt) and signal transducer and activator of transcription 3 (STAT3) in the presence of proinflammatory cytokines, especially IL-6 and IL-23, while Treg cells – by forkhead box protein 3 (Foxp3) transcription factor (Szandruk-Bender et al., 2022a; Szandruk-bender et al., 2023). Importantlythe gut microbiota directly or through metabolites indirectly can regulate Th17 and Treg cell differentiation and, thus, the progression of CD (Chen et al., 2023).

Intestine inflammatory response is also determined through the remodeling of the extracellular matrix by the action of upregulated metalloproteins, e.g., MMP1, MMP3, MMP9, and the overexpression of such adhesion. Its overexpression enables increased migration of lymphocytes to the healthy gastrointestinal tract and sites of inflammation (Petagna et al., 2020). Disturbances in the apoptosis process also contribute to CD pathogenesis. Excessively expressed apoptosis of epithelial cells leads to their increased elimination and damage to the intestinal barrier, that said reduced programmed death of inflammatory cellsresults in their accumulation in the wall of the gastrointestinal tract and maintenance of inflammatory process (Guan, 2019).

### 1.3 Genetic factors in the pathogenesis of Crohn’s disease

There is a growing body of evidence that genetic factors influence the risk of developing CD increasingly confirmed susceptibility loci for CD (Graham and Xavier, 2020). The first gene whose mutations were associated with this disease is nucleotide-binding oligomerization domain 2 (NOD2). NOD2 mutations occur in around one-third of the CD patients. The 1007fs mutation in this gene manifests itself in a more severe course of the disease, and the R702W and G908R mutations lead to an intensified response from proinflammatory cytokines and the induction of inflammation (Guan, 2019). In addition to *NOD2*, genes associated with the risk of developing CD are related to i) the innate pattern recognition receptors, e.g., caspase activating recruitment domain 15 (CARD15), organic cation transporters novel (OCTN), toll-like receptors (TLRs); ii) the integrity of the intestinal barrier, e.g., (DLG5, IBD5); iii) autophagy, microbial detection, and effector pathways, e.g., autophagy-related gene 16L1 (ATG16L1), immunity-related GTPase M (IRGM), leucine-rich repeat kinase 2 (LRRK2); and iv) lymphocyte differentiation, e.g., interleukin-23 receptor (IL23R), STAT3, ROR, TNFSF15, Janus kinase 2 (JAK2), chemokine receptor 6 (CCR6) (Tsianos et al., 2012; Graham and Xavier, 2020). Moreover, many genes appear to be not only susceptibility genes but also influence the prognosis (NOD2, IL23R, ATG16L1, DLG5, IRGM), disease activity (NOD2), location (ATG16L1, NOD2, IL-10, STAT3, TLRs) of CD, as well as the presence of intestinal and extraintestinal manifestations in the course of CD (TLRs, CARD15, NOD2, IL-6, IL-10, IRGM, STAT3) (Tsianos et al., 2012). Even though many people carry loci that increase the risk of CD, only a small proportion of the population develops CD. The occurrence of the disease requires exposure to environmental factors and disruption of the interaction between the intestinal microbiota and the immune system of the intestinal mucosa (Graham and Xavier, 2020).

### 1.4 Environmental factors in the pathogenesis of Crohn’s disease

Prenatal life: Development of CD in children is influenced by the mother’s age (>35 years old) and smoking during pregnancy (Roberts et al., 2011). Increased risk for IBD, including CD, also occurs after exposure to antibiotics during the 3rd trimester of pregnancy (Örtqvist et al., 2019).

Perinatal factors: The studies on these factors looked at prematurity, month of birth, birth weight, and Apgar score obtained. It was shown that only an Apgar score of.7 at one minute was associated with a higher probability of CD (Canova et al., 2020).

Neonatal and infancy period: Many case-control studies have shown an association of breastfeeding with later incidence of CD (Gruber et al., 1996; Basson et al., 2014). One study reported that CD patients lived in smaller households and had lower numbers of siblings (Bernstein et al., 2006). In addition, the study performed by Hampe et al. additionally showed that lower birth position is a possible indicator of increased exposure to infections, resulting in a higher risk of CD (Hampe et al., 2003). The place of living is also important. Numerous studies have shown that people who spent their childhood in the countryside have a much lower probability of developing CD in adulthood (Radon et al., 2007; Benchimol et al., 2017). Other factors related to childhood include the level of hygiene. Indeed, it has been proven a directly proportional relationship that the higher the level of hygiene, the greater the likelihood of CD (Amre et al., 2006; Lashner and Loftus, 2006).

Specialist risk factors in adult life: *1) Smoking*. Studies have shown that active smokers and ex-smokers have a significantly increased risk of CD compared to people who have never smoked (Lakatos et al., 2007; Berkowitz et al., 2018). It has been suggested that they are contributed to by i) the T-cell-nicotine connection (released immune messengers lead to intestinal inflammation) (Razani-Boroujerdi et al., 2007); ii) modifications of mucus production by the gastric mucosa (Li et al., 2014) and intestines (Allais et al., 2016); iii) disorders of the intestinal mucosal repair (Li et al., 2014) and iv) disruption of blood flow to the mucosa of the gastrointestinal tract (Hunsballe et al., 2001). *2) Supplementation of chemical substances*. Many studies have confirmed the impact of using antibiotics (Card et al., 2004; Hildebrand et al., 2008), and nonsteroidal anti-inflammatory drugs (Felder et al., 2000) on the development of CD. These compounds probably cause damage to the mucosa of the gastrointestinal tract,consequently disrupting the formation of the its microbiome. Moreover, the use of oral hormone therapy has been shown to be positively associated with the risk of CD, it has been proven that it is independent of the dose of estrogen used (Cornish et al., 2008). *3) Diet.* A high consumption of animal protein and long-chain omega-6 polyunsaturated fatty acids has been associated with an increased risk of CD (Shoda et al., 1996). It is due to the fact that omega-6 fatty acids are indirectly involved in the production leukotrienes and prostaglandins. *4) Other*. Exacerbation of symptoms in CD is also influenced by strong stress and sleep disturbances, which have been observed during periods of recurrence (Anthony Sofia et al., 2020; de Dios-Duarte et al., 2022).

### 1.5 Microbial factors in the pathogenesis of Crohn’s disease

In fact, many studies have shown that one of the most likely factors in CD is an imbalance in the gut microbiota. These changes contribute to the impairment of intestinal innate immunity carried out by neutrophils, monocytes, macrophages, dendritic cells, innate lymphoid cells, and natural killer (NK) cells, representatives of non-specific first-line defense. Furthermore, studies have shown that in the situation of impaired intestinal microbiota, intestinal CX3C chemokine receptor 1 high (CX3CR1^high^) macrophages differentiate into pro-inflammatory effector cells, acquiring the ability to present antigens to lymphocytes and becoming a critical predisposing factor in the development of IBD, including CD (Zigmond et al., 2014).

Explicit experimental evidence was provided by the studies of Schaubeck et al. (2016). In their study, they used a transplant of CD-associated microbiota that transferred features of colitis into the recipient’s body. This confirmed the direct causal role of intestinal bacterial dysbiosis in the development of chronic enterocolitis.

One theory regarding the etiology of CD points to the involvement of completely different microorganisms in the initiation of the disease than in its development. Types that represent a small percentage of the gastrointestinal microflora appear to be involved in the initiation. These include: *Proteobacteria* (e.g., *E. coli*, *Helicobacter* spp.) (Arumugam et al., 2011; Carrière et al., 2014), *Actinobacteria (e.g., Mycobacterium avium* subsp. *paratuberculosis)* (McNees et al., 2015; Elmagzoub et al., 2022), and also viruses (e.g., norovirus, polyomavirus, anellovirus, herpesvirus, adenovirus, sapovirus, rotavirus) (Haag et al., 2015; Lecuit and Eloit, 2017; Cao et al., 2022; Matsuzawa-Ishimoto et al., 2022; Dehghani et al., 2023; Ding et al., 2023) and fungi (e.g., *Candida* spp.*)* (Šašala et al., 2020; Di Martino et al., 2022). In contrast, the role in maintaining inflammation is mostly attributed to species of the genus *Firmicutes* and *Bacteroides*, which account for >90% of the total human intestinal microbiota (Frank et al., 2007; Sokol et al., 2008; Mondot et al., 2011). Studies have shown that sustained inflammation is associated with a reduction in the amount of *Faecalibacterium prausnitzii* and *Bacteroides fragilis* and an increase in *E. coli* and mucolytic bacteria: i.e., *Ruminococcus gnavu*s and *Ruminococcus torques* (Darfeuille-Michaud et al., 2004; Sokol et al., 2008; Png et al., 2010). Due to the fact that no microorganism has been isolated that is present in all patients with CD, the direct role of microorganisms in the progression of the disease has not been determined. Therefore, the contribution of dysbiosis is highly probable. The conducted studies confirm that dysbiosis is a cause and also an effect of CD. In the early stages of the disease, a significantly reduced diversity of bacteria belonging to the intestinal microbiota is observed The aggressive groups (i.e., *Proteobacteria* spp., *Fusobacterium* spp. and *R. qnavus*) are dominant, developing at the expense of protective groups (i.e., *Lachnospiracea*e spp.*, Bifidobacterium* spp., *Roseburia* spp. and *Sutterella* spp.) (Sartor and Wu, 2017).

Confirmed decreased numbers of bacteria from the *Bacteroidales* family contribute to lower control of our immune system during infection. This is because this family is the main producer of mucins, the glycoproteins that make up mucus, which plays a protective and uptake role against pathogens (Szewczyk et al., 2019). As a result, the gut becomes more susceptible to infection.

Furthermore, disorders in the intestinal microbiota lead to increased levels of zonulin, a protein responsible for controlling the permeability of the intestinal barrier (Sturgeon and Fasano, 2016; Ohlsson et al., 2017). Increased amounts of this protein lead to disturbances in the integrity of the tight junctions between enterocytes, consequently contributing to the leaky gut syndrome. Bacteria of the *Ruminococcaceae* family, whose increasing amounts have been confirmed in the progression of CD, are also involved in this process. These bacteria produce secondary bile acids, promoting the overproduction of reactive oxygen species (ROS), thereby reducing the integrity of the intestinal barrier (Hang et al., 2022).

An increase in the number of *Ruminococcus* spp. (i.e., *R. gnavus, R. torques*) may also contribute to an abnormal response from the immune system. Indeed, these bacteria produce short-chain fatty acid (SCFA), thus playing a huge role in the body’s immune system response, including regulating the production of cytokines (Igudesman et al., 2022).

On the other hand, *B. fragilis* whose decline has been documented in the progression of CD, secretes lipopolysaccharide (LPS), which activates the transcription nuclear factor kappa light chain enhancer of activated B cells (NF-κB). This factor plays a significant role in immune and inflammatory processes, as it regulates the expression of many genes, including those associated with the production of cytokines, acute-phase proteins, collagenases, stromilysins, and matrix-degrading enzymes. Moreover, it demonstrates the ability to inhibit apoptosis, induce proliferation, and enhance the angiogenesis process, suggesting its involvement in the processes of oncogenesis and tumor progression (Kou et al., 2020). Furthermore, NK-κB is responsible for inducing the transcription of microRNA (i.e., miRNA-9, miRNA-34, miRNA-125b, miRNA-146a, miRNA-155) with proinflammatory effects. Additionally, it activates miRNA-34a, which inhibits the expression of the triggering receptor expressed in myeloid/microglial cells (TREM) (Bhattacharjee et al., 2016; Lukiw et al., 2021). Accordingly, this contributes to the disruption of the microglia’s anti-phagocytic abilities, promoting neuroinflammatory diseases. Therefore, the reduction in the abundance of *B. fragilis*, and consequently a decrease in the LPS produced by these bacteria, contributes to the development of inflammatory and neoplastic diseases.

Moreover, bacteria belonging to the gastrointestinal microbiome adapt to participate in diseases with coexisting genetic, environmental, and immunological conditions. They do this not only by influencing mucus components and tight junctions but also by producing adhesins. One of these are curli fimbriae, which exhibit the biochemical and structural properties of β-amyloid (Aβ) (Sobieszczańska et al., 2019). This protein is a constituent of various healthy tissues, including the heart, muscle, liver, kidney, and brain. However, under favorable conditions, it can also become pathogenic in these tissues (Sonthalia et al., 2016; Martínez-Naharro et al., 2018; Pinto et al., 2021; Deng et al., 2022; Gurung and Li, 2022). Furthermore, previous studies have confirmed that human amyloid and bacterial amyloid share many common features, undoubtedly warranting a more in-depth analysis (Das et al., 2022; Bessho et al., 2023).

It is evident that dysbiosis within the gastrointestinal tract opens the gates of the intestines to toxins and proinflammatory cytokines, thereby likely increasing the probability of inflammatory diseases (including CD), and, consequently, the development of neurodegenerative diseases, i.e., Alzheimer’s disease (AD).

However, it is a known fact that the functions attributed to the intestine, i.e., immune activation, intestinal permeability, its reflexes, and enteroendocrine transmission, are controlled by the gut-brain axis (GBA) (Carabotti et al., 2015). GBA plays a significant role in shaping both the structure and development of the central nervous system (CNS) (Foster et al., 2017). This communication between the gastrointestinal tract and the CNS occurs bidirectionally: through indirect and direct pathways, involving neuronal, humoral, and immunologic paths (Montiel-Castro et al., 2013). Numerous studies conducted in recent years have shown that the role of gut microbiota goes far beyond functions related to the digestive system. It influences, among other things, the immune system, carbohydrate metabolism, bone health, and also plays a crucial role in connection with GBA (Anglin et al., 2015). Experiments performed on animals have provided valuable insights into this topic. Indeed, it has been demonstrated that intestinal colonization is essential for the proper development of the CNS. On the other hand, the absence of gut microbiota in the studied animals clearly affected the disturbance of neurotransmitter expression and activity, thereby disrupting the functioning of the CNS. This manifested as memory problems, the development of anxiety, and depressive disorders (Carabotti et al., 2015). The enteric nervous system and the brain are in constant communication, and their interaction is made possible through the gut-microbiota-brain axis (GMBA).

## 2 Properties of amyloid protein

**Figure 1:**
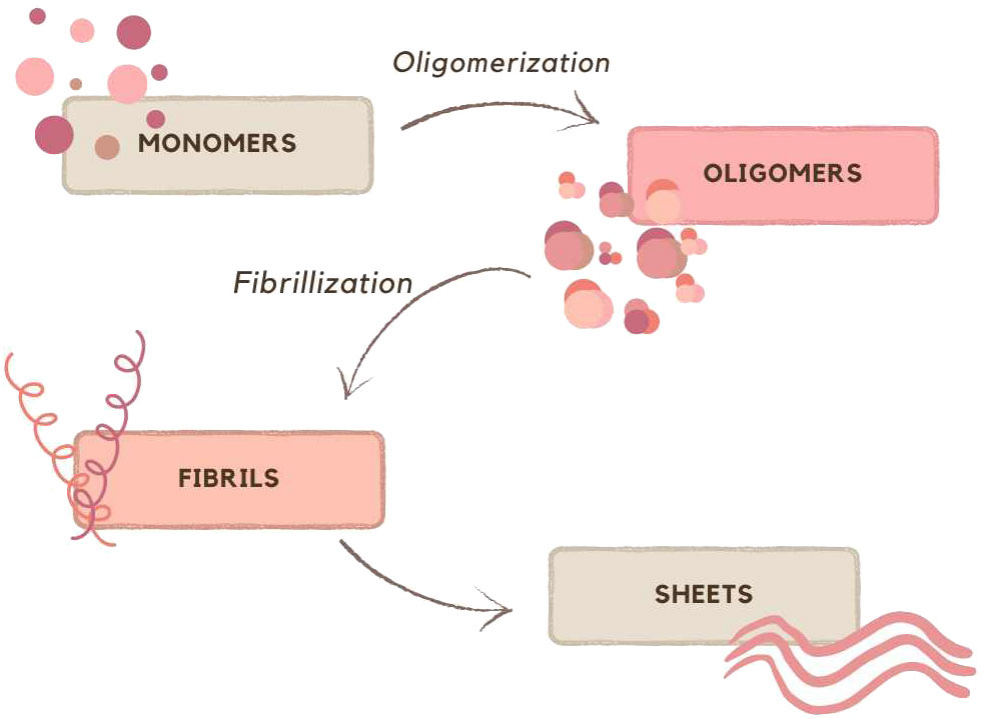
Schematic representation of formation sheets; based on (Bissig et al., 2016).

Amyloids are a diverse group containing many different proteins, which have in common β-sheet structures that aggregate into fibers. Their assembly starts from a monomer that oligomerizes and assembles into fibrils, which then organize into sheets (Bissig et al., 2016), are shown on **Figure 1**.

The AmyloGraph is comprehensive database highlighting interactions between 46 amyloid proteins or peptides (Burdukiewicz et al., 2023). According to the collected data, Aβ can alter fibrillization speed (faster/slower aggregation) on both the same or another amyloid which can also result in the heterogenous fibers. This can have positive and negative effects on the human body. Aβ contributes in various disease and important pathways, and understanding these interactions may be helpful in the prevention and management of them. Atrial natriuretic peptide (ANP) through a process called ‘cross-seeding’ inhibits Aβ aggregation (Tang et al., 2023). Gelsolin responsible for Finnish type of familial amyloidosis just like ANP inhibits the fibrilization of Aβ (Maury, 1991; Ray et al., 2000). In **Table 1** are presented several protein capable of amyloid formation, diseases caused by them and its functions. Some of them are neurodegenerative diseases, e.g., Parkinson’s disease (PD), AD and prion disease, e.g., Creutzfeldt-Jakob disease (CJD), fatal familial insomnia (FFI) thus the concept that tumors are prion like disease is reflected: S100A9, p53, Beta-2-microglobulin (B2M) amyloids are involved in numerous tumors (Li et al., 2022). Moreover, Aβ can be both positive and negative for our health, yet there is still vast advantage for harmful aspects.

**Table 1:** Contribution of amyloid protein to disease pathogenesis.

Protein	Disease	Function	Source
α-synuclein	Parkinson’s disease	intracellular and synaptic vesicle trafficking	(Villar-Piqué et al., 2016; Mehra et al., 2019)
Aβ	Alzheimer’s disease	neurite growth, neuronal adhesion	(Baumkötter et al., 2014; Trejo-Lopez et al., 2019)
Apolipoprotein A-I	Hereditary AApoAI	formation of HDL, lipid transport	(Traynor et al., 2013; Frankel et al., 2022)
Cystatin C	HCCAA	inhibitor of cysteine proteinases	(Osk Snorradottir et al., 2015; Ding et al., 2022)
IAPP	Diabetes type II	regulator of energy metabolism	(Wiltzius et al., 2009; Hay et al., 2015)
Insulin	Insulin amyloidosis	lowers glucose level, anabolic hormone	(Tokarz et al., 2018; Kano, 2022)
Lysozyme	OTA	bacteriolytic function	(Pepys et al., 1993; Wu et al., 2019)
Pmel 17	NN	melanosome morphogenesis, pigmentation	(Bissig et al., 2016)
proSP-C	chILD	lowers surface tension in alveolars	(Griese et al., 2016; Barriga et al., 2021)
PrP	CJD, FFI, GSD, HDL1	neuronal development and synaptic plasticity	(Taylor et al., 2009)
S100A9	tumor development	Ca^2+^;Zn^2+^ binding protein	(Marinković et al., 2020)
Serum amyloid A	PCOS	acute-phase response	(Sun and Ye, 2016; Liu et al., 2022)
p53	tumors, cancer, LFS	tumor suppressor in many tumor types	(Li et al., 2022)
B2M	breast cancer, RCC	tumor-promoting and tumor-suppressing	(Wang et al., 2023)
Tau	FTD	promotes microtubule assembly and stability	(Yoshida and Goedert, 2012)
TDP-43	ALS	RNA-binding protein	(Bhardwaj et al., 2013; Yu et al., 2020)
Transthyretin	ATTR-CM	thyroid hormone-binding protein.	(Ruberg et al., 2019)

Aβ, β-amyloid; IAPP, islet amyloid polypeptide; Pmel 17, premelanosome protein 17; proSP-C, prosurfactant protein C; PrP, prion protein; S100A9, S100 calcium-binding; protein A9; p53, regulatory protein; B2M, beta-2- microglobulin; TDP-43, TAR; DNA-binding protein 43; AApoAI, Apolipoprotein AI-derived amyloidosis; HCCAA, Hereditary Cystatin C Amyloid Angiopathy; OTA, Ostertag-type amyloidosis; NN, not named; chILD, children’s interstitial lung disease; CJD, Creutzfeldt-Jakob disease; FFI, fatal familial insomnia; GSD, Gerstmann-Straussler disease; HDL1, Huntington disease-like type 1; PCOS, polycystic ovary syndrome; LFS, Li-Fraumeni syndrome; RCC, renal cell carcinoma; FTD, Frontotemporal dementia; ALS, Amyotrophic lateral sclerosis; ATTR-CM, Transthyretin Amyloid Cardiomyopathy; HDL, high-density lipoprotein

## 3 Amyloids in neurodegenerative disorders

**Figure 2:**
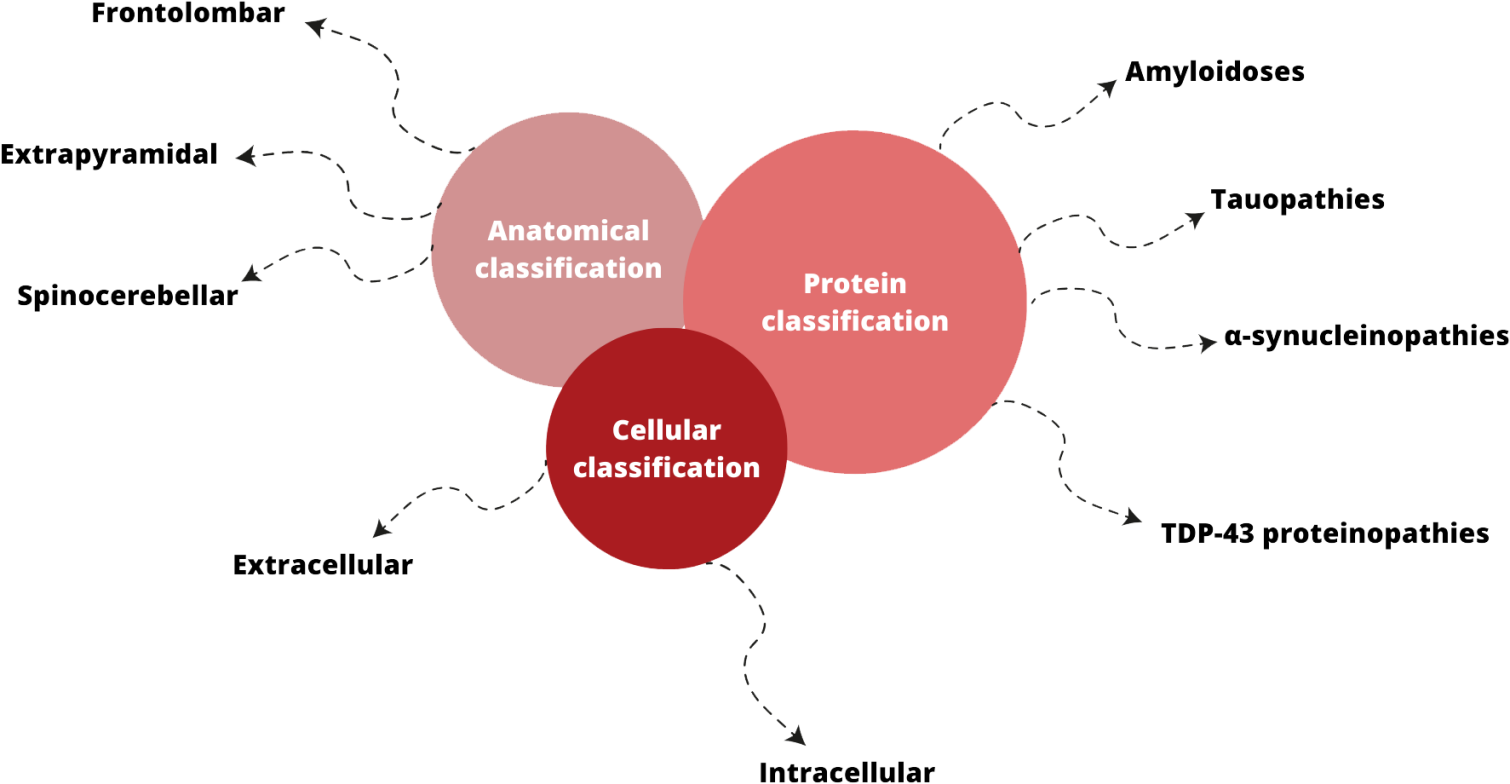
Classifications of neurodegenerative disorders; based on (Kovacs, 2016; Kovacs, 2018; Sahoo et al., 2022).

Neurodegenerative disorders (ND) affect millions of people in the world and seem to become one of the greatest global health problem (Van Schependom and D’haeseleer, 2023). Classification of ND can be based on anatomical, cellular ground or according to type of amyloid involved (Kovacs, 2016; Kovacs, 2018) (**Figure 2**). Anatomical classification emphasizes the affected regions in the neural system and, as a result of this localization, the clinical manifestations. On the other hand, the cellular classification focuses on molecular pathology and distinguishes whether amyloid deposits accumulate intracellularly or extracellularly (Kovacs, 2016).

Although exact causes of these diseases are still unknown, their pathomechanism is associated with misprocessing of proteins that aggregate and accumulate in neural tissue (Wolfe and Cyr, 2011; Wells et al., 2021). The factor that is responsible for this proteostasis dysfunction is unidentified, yet there are theories about possible processes which can lead to abnormal protein aggregation, for example oxidative stress, mitochondrial disfunction or neuroinflammation (Bonafede and Mariotti, 2017; Alexander, 2004). Although the pathomechanism of many of these diseases has not yet been fully clarified, some of them have been the subject of many studies aimed at explaining them. These include PD, amyotrophic lateral sclerosis (ALS), and Huntington’s disease (HD), the pathomechanism of which is briefly discussed below.

Parkinson’s disease, second most common neurodegenerative disorder, develops when α-synuclein forms intracellular aggregates, Lewy’s bodies, in dopaminergic neurons of the *substantia nigra* (Sveinbjornsdottir, 2016; Kouli et al., 2018; Alexander, 2004). It leads to loss of neurons and decreased levels of dopamine that is responsible for clinical symptoms like bradykinesia, rigidity, tremor, and balance problems (Sveinbjornsdottir, 2016; Kouli et al., 2018).

**Figure 3:**
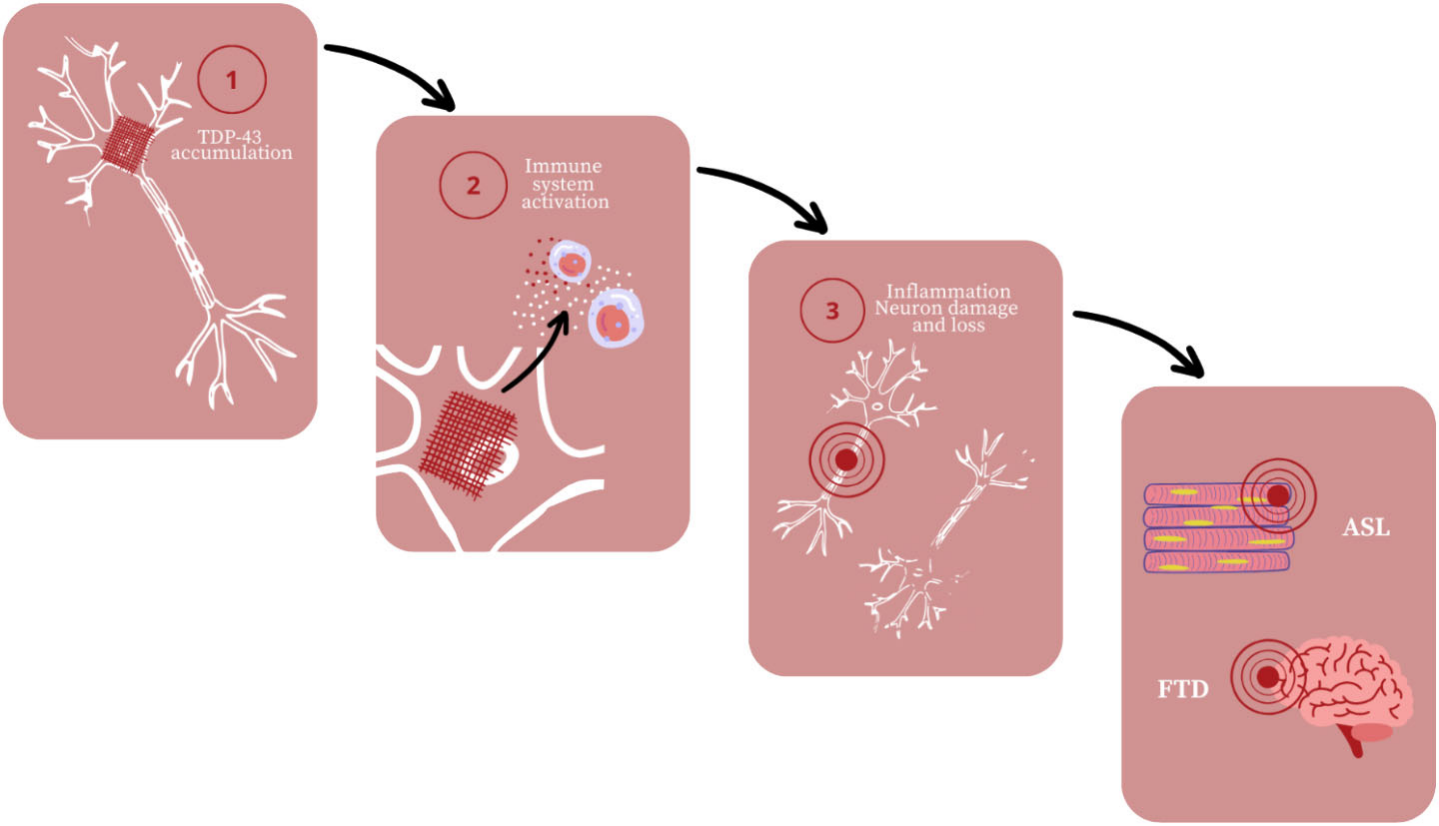
Amyotrophic lateral sclerosis (ALS) and fronto-temporal dementia (FTD) development; based on (Yu et al., 2020).

Among neurodegenerative diseases there are also motor neuron diseases that affect motoneurons and cause muscle paralysis (Motor neuron diseases; Bonafede and Mariotti, 2017). Amyotrophic lateral sclerosis is the most common out of these diseases though new evidence reveal that ALS is a multisystem disorder (Mishra et al., 2020; Matrone, 2023). Not only it attacks both upper and lower motor neurons but also non-motor structures what results in fronto-temporal dementia (FTD) (Mishra et al., 2020; Mahoney et al., 2021). In conclusion, ALS cause progressive muscle paralysis and behavioral, language, cognition changes (Mishra et al., 2020; Mahoney et al., 2021). The factor that is considered to play a role in the pathogenesis of ALS is mutated TAR DNA binding protein 43 (TDP-43) (Yu et al., 2020) (**Figure 3**).

However, TDP–43 may not be the only one amyloid protein that is involved in ALS pathogenesis. According to Bryson et al. onset of ALS symptoms coincide with increase of Aβ and amyloid precursor protein (APP) in muscles (Zhang and Shi YD, 2022). Although there are no evidences that these amyloids aggregation causes ALS the altered levels of them were observed in ALS patients, so it may be worth considering in future studies (Nishikawa et al., 2021).

Huntington’s disease is an autosomal dominantly inherited, late-onset, polyglutamine, neurodegenerative disorder (McGowan et al., 2000; Churkina et al., 2022). It is a result of the expansion of trinucleotide cytosine–adenine–guanine (CAG) in *HTT* gene that causes formation of mutated protein huntingtin (Churkina et al., 2022). Because of its deformed structure, huntingtin aggregates inside the neurons and causes dysregulation in cell’s processes such as protein degradation, mitosis or signaling pathways (Matlahov and van der Wel, 2019; Churkina et al., 2022). It leads to neurons death and manifest as uncontrolled movement, abnormal behaviour, changes in personality and emotions (Huntington’s disease).

The great breakthrough has been made in the field of amyloid diseases as scientists from the Stowers Institute for Medical Research have uncovered the structure of the first step in Aβ formation for Huntington’s disease (Stowers Institute; Kandola et al., 2023). It may give new prospects for HD treatment and reveal some secrets of amyloids.

All things considered, NDs form diverse group of diseases that may vary in pathomechanism or location of leasions, but have common thread – amyloids. Aβ, among all of amyloid proteins, seems to play a significant role in many of these disorders and is noteworthy in future research.

## 4 Contribution of amyloid protein to the pathomechanism of Alzheimer’s disease

Neurodegenerative diseases, including AD, can be called proteinopathy. Aggregated extracellular Aβ plaques and intracellular Tau protein (Tau) tangles are well-known protein pathologies of AD. Increasing evidence suggests that the development of AD characteristic pathological features, i.e., β-amyloid plaques and Tau tangles, can be associated with microorganisms (Dow, 2021).

Alzheimer’s disease, a neurodegenerative disorder, is most common dementia, possibly contributes to 60-70% of cases worldwide which is over 30 million people with AD according to the World Health Organization (Zhang et al., 2021).

### 4.1 AD’s pathology

Pathophysiology of AD is very complex, main contributing factors are: genetics, epigenetics, microbiota, immunology and environment. It is based on many known mechanisms of neurodegeneration, including dysregulation of calcium homeostasis, abnormal accumulation of Aβ and dysfunctional Tau, imbalance of neurotransmitters, necrotic and apoptotic neuronal death, disappearance of synapses, and neuroinflammation with pathological microglia and astrocyte activation in the brain, white matter changes and finally brain atrophy (Pluta et al., 2020). AD is divided into 2 subtype: early-onset Alzheimer’s disease (EOAD), defined as Alzheimer’s disease occurring before age 65 and late-onset Alzheimer’s disease (LOAD). LOAD is more frequent, thus well studied and usually is more mild progressive (Tellechea et al., 2018; Perkovic et al., 2021).

### 4.2 Genetic factors

The amyloid cascade hypothesis is based on Aβ accumulation resulting in the initiation of a cascade leading to neurodegeneration. The integral genes contributing to this process are *APP*, presenilin 1 (*PSEN1*), and presenilin 2 (*PSEN2*) genes (Reitz, 2015). These genes affect amyloid production or cleavage and are primarily involved in the EOAD. The development of LOAD is more complex and those mutations are not mandatory. The basic principle of overproduction and/or impaired clearance of Aβ stays the same for both EOAD and LOAD yet the pathways are different (Robinson et al., 2017). The apolipoprotein E (APOE) was first genetic risk factor for LOAD. Its presence determines increased risk of AD, accelerating symptoms and lowers the age at onset by 6-7 years (Reitz, 2015; Robinson et al., 2017). Also, *APOE4* and *TREM2* genes are involved in AD, being responsible for cholesterol metabolism and immune response, respectively (Karch and Goate, 2015).

### 4.3 Epigenetics

Epigenetics play a major role in the development, diagnosis and therapy of AD (Perkovic et al., 2021). The epigenetic alterations in AD include: DNA methylation/hydroxymethylation, mitochondrial DNA (mtDNA) methylation, histone modifications, the microRNAs. Results collected from other work by Perkovic et al. suggest involvement of 5mC (5-methyl cytosine) and 5hmC (5-hydroxymethyl cytosine) in AD pathology and progression. These compounds are products of cytosine methylation leading to weaker binding of transcriptional factors. Authors point out its difficult to compare results from experiments using different methods (methylation array technology, next generation sequencing, and pyrosequencing and immunochemistry) and different brain regions tissues. miRNAs significant in pathology of AD have biomarker potential as easily monitored in body fluids, their level can be used as distinction from other dementias as AD does not have common detecting test. Lastly mtDNA also may be potential marker (Perkovic et al., 2021). The dysregulation of DNA methylation dynamics, encompassing both hypermethylation and hypomethylation events, contributes to the disruption of transcriptional programs underlying synaptic plasticity, neuroinflammation, and Aβ deposition, thereby exacerbating the neurodegenerative cascade characteristic of AD (Maity et al., 2021; Sommerer et al., 2023). Histone modifications, encompassing an array of reversible post-translational alterations to histone tails, exert fine-tuned control over chromatin accessibility and gene expression. Perturbations in histone acetylation, methylation, and phosphorylation have been implicated in AD pathophysiology, modulating the expression of genes central to neuronal survival, synaptic integrity, and cognitive function (Anderson and Turko, 2015; Santana et al., 2023). Notably, the dysregulation of epigenetic enzymes, including DNA methyltransferases and histone-modifying enzymes, underscores the intricate interplay between genetic and epigenetic factors in AD susceptibility and progression. Targeting epigenetic modifiers presents a tantalizing avenue for therapeutic intervention, with epigenetic-based therapies poised to mitigate the progression of AD pathology and ameliorate cognitive decline.

### 4.4 Microbiota

**Figure 4:**
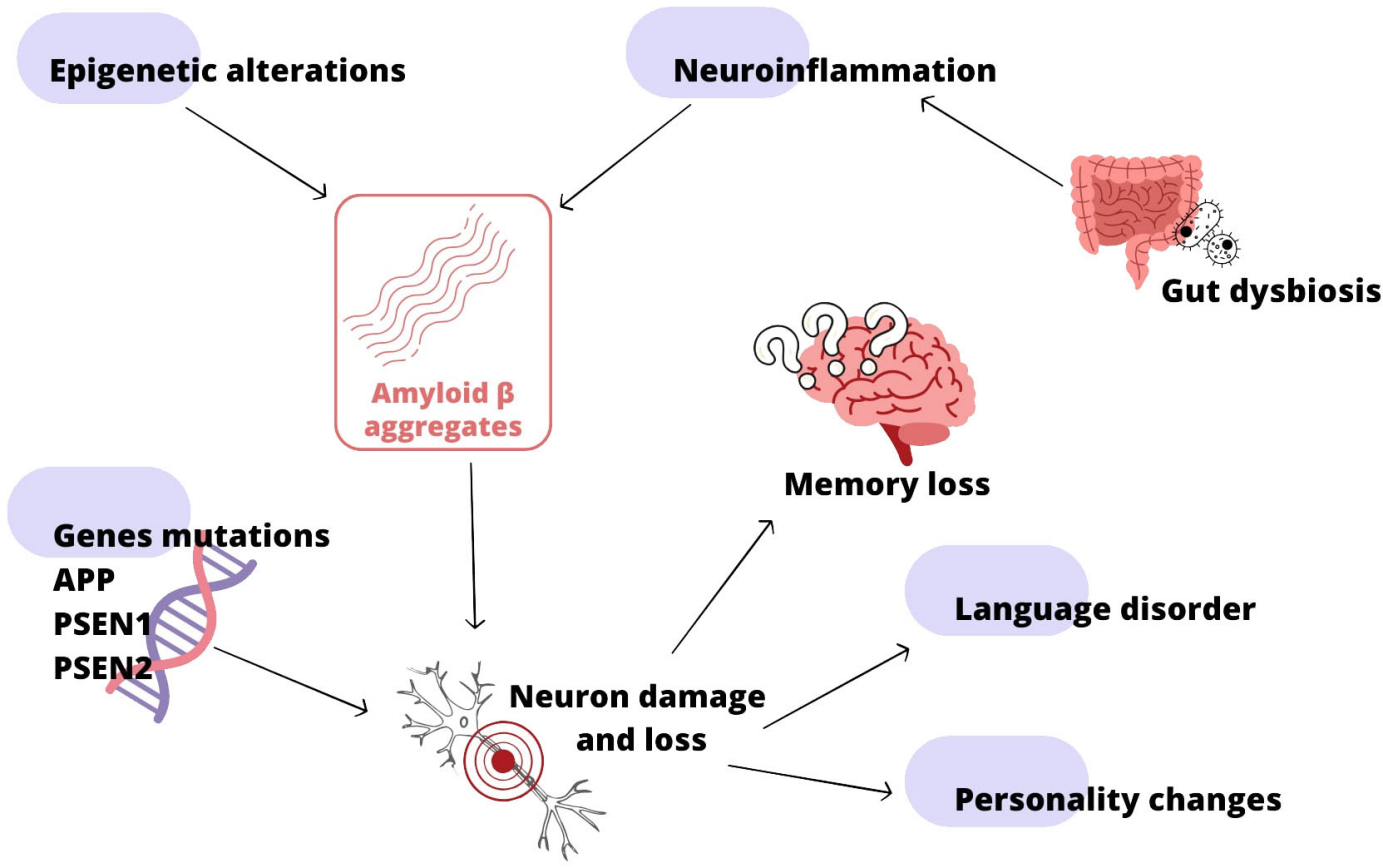
Alzheimer’s disease (AD) development; based on (Reitz, 2015; Moir et al., 2018; Megur et al., 2020; Pluta et al., 2020; Perkovic et al., 2021).

The growth of microbiota starts even before birth of the child, crucial for development is the first year of age, nevertheless its state is dynamic until a person dies with it (Gomaa, 2020; Vandenplas et al., 2020). That gives us opportunity to maintain it during life time. Microbiota is element of the GMBA capable of altering mood, behavior and other processes through immune, neuroendocrine and direct nerve mechanisms. Its role is multi-level, over the last years scientists discovered many connections between some illnesses and microbiota, e.g., HIV, obesity, allergies and many other (Desai and Landay, 2018; Vandenplas et al., 2020). Changes in microbiota can cause anxiety, memory impairment, cognitive and neurodegenerative disorders (Pluta et al., 2020). As indicated above the intestinal dysbiosis is the source of Aβ, LPS and other toxins, which contribute to systemic inflammation and disruption of physiological barriers, e.g., intestinal wall (Megur et al., 2020). These products can transfer, through X cranial nerve, to CNS over years, triggering inflammation and microglia activation. Neuroinflammation is the reason of neuron loss in the brain. Combined with bacterial amyloid it promotes misfolding and aggregation of human amyloids (Megur et al., 2020; Pluta et al., 2020). There is hypothesis of antimicrobial protection in AD. According to theory Aβ deposition is an early immune response to mistakenly perceived immunostymuli. Aβ fibrillization helps to combat the infection, in AD, chronic activation of this pathway leads to sustained inflammation and neurodegeneration (Moir et al., 2018). Therefore the regulation of microbiota shines like a prominent opportunity to manage AD. It is possible mostly through healthy diet rich in fibers, yet also probiotics and antibiotics have its role. The effect of fructants on reducing AD incidence in the elderly, among other things, has been demonstrated (Nishikawa et al., 2021). By appropriate distribution of some antibiotics: amoxicillin, minocycline, rapamycin D-cycloserin, doxycycline it is possible to improve cognition, reduce Tau, Aβ, inflammation and microglia activation. However antibiotics: streptomycin, ampicillin, cefepime have negative impact on the animals and humans with AD (Angelucci et al., 2019). In the AD rat model administration of *Lactobacillus plantarum MTCC 1325*, *Lactobacillus* spp. *and Bifidobacterium*, *Bifidobacterium breve strain A1* have had positive impact on Aβ formation or its effects on cognitive functions (Megur et al., 2020). The pathways to the development of Alzheimer’s disease are shown in **Figure 4**.

As there is connection between gut microbiota and ND development and progression, alterations in the intestinal microbial flora may be promising treatment option. Akbari E et al. investigated effect of probiotic supplementation in patients with AD (Akbari et al., 2016). During randomized, double-blind, and controlled clinical trial, treatment group of patients supplemented probiotic milk containing *Lactobacillus acidophilus, Lactobacillus casei, Bifidobacterium bifidum* and *Lactobacillus fermentum* for 12 weeks (Maity et al., 2021). Similar trial was conducted by Tamtaji et al., as patients were aministered probiotic containing *Lactobacillus acidophilus*, *Bifidobacterium bifidum*, and *Bifidobacterium longum* with selenium for 12 weeks (Tamtaji et al., 2019). Both trials showed positive effect of probiotics on cognitive function and some metabolic profiles in AD (Maity et al., 2021; Santana et al., 2023). Beneficial effect on AD appears to be treatment involving fecal microbiota transplantation (FMT) (Xiang et al., 2023). *In vivo* studies conducted by Soriano et al. in the C57BL/6 mouse model confirmed the roles of the intestinal microbiota in the pathogenesis of AD. Healthy mice treated with fecal microflora from mice with AD revealed larger areas of brain damage, increased numbers of activated microglia cells and reduced motor regeneration (Soriano et al., 2022). These studies provide the basis for the hypothesis that it is the microbiota that can improve cognitive function in NDs and improve recovery. These optimistic results create basics for further studies in this area.

## 5 Crohn’s disease association with Alzheimer’s disease

Extensive research in recent years has provided incontrovertible conclusions that the gut microbiota is closely linked to neurodegenerative diseases. Increasing evidence suggests that the gastrointestinal tract plays a meaningful role in AD. Moreover, patients with CD are at an increased risk of developing AD (Wang et al., 2022). Previous studies confirm that the pathophysiological mechanisms leading to gastrointestinal disorders terminally lead to neurodegeneration. A meta-analysis concerning residents of Taiwan, conducted by Liu et al., showed that the likelihood of dementia occurrence in people with IBD, including CD was twice as high. Furthermore, the risk of developing AD was the greatest among all types of neurodegenerative diseases (six times higher in people with vs without IBD). These conclusions are supported by a meta-analysis conducted by Szandruk-Bender et al., based on the search of Pubmed and Embase databases (Szandruk-Bender et al., 2022b).

### 5.1 Chronic inflammatory bowel disease is associated with increased inflammation of the nervous system

In recent years, numerous meta-analyses have been conducted to achieve consensus on the relationship between IBD and neurodegenerative diseases. Research by Zhang et al. provided evidence of potential dementia indicators in the course of IBD. They demonstrated that the risk of developing AD in patients with CD was twice as high [risk ratio (RR) of 2.79] compared to the general population [(RR) = 1.35] (Zhang and Shi YD, 2022). Furthermore, significant evidence was provided by Kim et al. in studies conducted among the Korean population. It was shown that in patients with IBD aged ≥ 65 years, the risk of AD was increased compared to the control group [adjusted hazard ratio (HR) = 1.14] (Kim et al., 2022).

Research by Heston et al. suggests that inflammatory bowel disease is linked to brain inflammation even in the early stages of the disease. These authors demonstrated that inflammation of the intestines may exacerbate the progression of AD. Their study involved measuring calprotectin, a marker of intestinal inflammation, in the feces of people with confirmed AD. The obtained results were subjected to multiple regression analysis with maximum likelihood estimation and Satorra-Bentler correlations, using 11C-Pittsburgh compound B positron emission tomography (PiB-PET) imaging, and synchronized with cognitive test results. It was shown that in patients diagnosed with AD, the level of calprotectin was higher. Furthermore, it also exhibited a higher level in those with impaired verbal memory functions but normal cognitive functions, thus indicating a very early stage of AD (Heston et al., 2023). Elevated levels of calprotectin observed in neurodegenerative diseases are also associated with its elevation in the course of CD (Bourgonje et al., 2018; Kennedy et al., 2019).

Additionally, studies performed by Liu et al. suggested that chronic inflammation combined with an abnormal gut microbiome may degrade cognitive functions proportionally to the duration of this condition. Indeed, the longer the period of IBD, the greater the risk of dementia development (Liu et al., 2022).

Research conducted by Kaneko et al. using *in vivo* studies in wild-type mouse models and the AD mouse model, *App^NL-G-F^*, demonstrated the involvement of the immune system in AD and CD. Upon inducing intestinal inflammation (using 2% dextran sodium sulfate, DSS), an increase in Aβ accumulation was observed in the brains of mice exhibiting AD-like symptoms. Through detailed single-cell RNA sequencing analysis (scRNA-req), a significant presence of neutrophils in the brains of these animals was identified. Furthermore, the administration of antibodies inhibited the polymerization reaction. These studies clearly indicate that neutrophil infiltration in the AD-altered brain is associated with the progression of intestinal inflammation (Kaneko et al., 2023). This occurs because neutrophils activated by microglia can cross the BBB and positively respond to Aβ aggregation, leading to the production of inflammatory cytokines (Park et al., 2019).

### 5.2 Dysbiosis of the intestinal microbiota

The above data point to the critical role of the gut microbiota in CD, implicating it in the development of AD. It is very interesting that studies conducted to date have shown that bacteria belonging to the same families and even species are involved in both CD and AD. Patients with AD have been shown to have significant changes in the intestinal microbiota composition for many types of bacteria (Ferreiro et al., 2023). The most significant changes involve genera: *Firmicutes*, *Bifidobacterium, Actinobacteria, Eubacteria*, and *Bacteroidetes*, as well as *E. coli, Shigella* spp., and *Salmonella* spp (Vogt et al., 2017). In addition, meta-analyses by Hung et al. involving patients with AD *vs* a control group showed increased amounts of *Proteobacteria, Firmicutes, Clostridiaceae, Lachnospiraceae* and *Rikenellaceae* in the AD spectrum group (Hung et al., 2022). Research on the gut microbiome in the course of CD and AD has revealed a decrease in the levels of *Prevotellaceae*, *Firmicutes*, *Actinobacteria*, and *Eubacterium*. This situation leads to the disruption of mucin synthesis and tight junctions between enterocytes. Consequently, it contributes to an increased permeability of the intestinal mucosal membrane (Paray et al., 2020; Socała et al., 2021). As a result, there is the occurrence of leaky gut syndrome, which is characterized not only by disruptions in the integrity of tight junctions but also by a decrease in the level of immunoglobulin A. Together, they constitute the first line of defense of the gastrointestinal tract against pathogens, a defense that is clearly compromised in the course of neurodegenerative diseases (Maruya et al., 2013). On the other hand, an increase in the quantity of bacteria from the *Ruminococcus* genus is associated with the production of secondary bile acids. This leads to DNA damage and an overproduction of ROS (Hang et al., 2022). It is known that ROS are a key harmful factor influencing the pathogenesis of neurodegenerative diseases, including AD. Simultaneously, oxidative stress leads to persistent damage to brain cells and disrupts the conduction of nerve impulses (Manoharan et al., 2016; Bhatt et al., 2021). As the involvement of the same bacteria has been confirmed in CD, the results of this research have directed scientists to make an effect-causal connection between diseases involving the gut and neurological diseases.

### 5.3 Decreased production of anti-inflammatory metabolites and increased bacterial neutotoxic and neuromodulatory molecules

Numerous studies conducted in recent years have provided interesting insights. They have demonstrated the direct involvement of bacterial products (such as proteins and metabolites) in the pathogenicity of the GMB-amyloid-AD connection, resulting from endothelial dysfunction (Marizzoni et al., 2020). Due to increased permeability of the gut-blood barrier, BBB, and GBA in progressing neurodegenerative diseases, the penetration of small particles such as amyloids, cytokines induced by LPS, or other small pro-inflammatory molecules is observed (Zhu et al., 2022). Confirmation of this phenomenon comes from studies conducted by Gonzalez Cordero et al., who analyzed eight observational experiments involving patients diagnosed with AD or PD. The results of their analysis clearly point to disruptions in the GBA, indicating a link between gut microbiota and cognitive dysfunction (Cordero et al., 2022). Furthermore, the direct involvement of bacterial factors in neurodegenerative diseases has been confirmed by researchers who have shown the impact of extracellular bacterial DNA (including its presence in the bloodstream) on the misfolding of Tau and the aggregation of Aβ (Tetz et al., 2020; Tetz and Tetz, 2021; Giacconi et al., 2023).

Other evidence linking the development of CD to changes occurring in patients with AD is the reduction in the quantity of *Bifidobacterium* spp. This genus actively participates in brain metabolic processes, including the production of the neurotransmitter gamma-aminobutyric acid (GABA). The level of this neurotransmitter in the gut nervous system correlates with its level in the central nervous system. Therefore, a decrease in the number of *Bifidobacterium* spp. species may be a factor contributing to changes characteristic of AD, such as the development of depression or abnormal cognitive functions (Chen et al., 2021).

Intestinal dysbiosis leads to a reduction in the production of beneficial anti-inflammatory metabolites by the intestinal microbiome, such as SCFAs, certain bile acids (e.g., tauroursodeoxycholic acid), and ligands for the aryl hydrocarbon receptor. These substances have the ability to traverse the BBB. On the other hand, chronic inflammation in IBD promotes the production of neurotoxic metabolites that contribute to inflammation within the nervous system. These neurotoxic metabolites include kynurenine, certain bile acids, LPS, and enterotoxins. They damage the lining of the large intestine, increasing its permeability. “Leaky, damaged intestines” serve as gateways for the migration of these metabolites from the intestinal lumen to the central nervous system, crossing through the GBA pathway (Jia et al., 2020; Mulak, 2021; Wang et al., 2022).

### 5.4 Association of amyloid (including curli fimbriae) with the development of neurodegenerative diseases

One link between neurodegenerative diseases and gastrointestinal diseases is the fact that some proinflammatory bacteria, such as *E. coli, Shigella* spp., *Salmonella* spp., and those of the genus *Bacteroidetes*, which growth has been demonstrated in both CD and AD, have the ability to produce amyloid peptides. These contribute directly to the development of AD. Furthermore, studies have shown that bacterial amyloid peptides (curli) structurally similar to amyloid fibers deposited in the brain in the form of plaques during AD (Miller et al., 2021). Previous studies have shown that curli fibers are an important factor in facilitating the bacteria that produce them to successfully colonize the colonic epithelium in people with IBD (including CD) (Sobieszczańska et al., 2019). Therefore, bacterial amyloids potentially affect amyloid aggregation in the brain and inflammation of the nervous system (Friedland and Chapman, 2017). Additionally, curli fibers have a very specific protective role for bacteria against external factors. They form “nets” preventing an effective response from the immune system. Moreover, the accumulation of Aβ is associated with the formation of neurofibrillary tangles composed of hyperphosphorylated Tau. This phenomenon is exactly one of the most characteristic changes in the course of AD. Indeed, it has been shown that there can be two *vs*. eight or more phosphoryl groups per molecule of Tau, for a healthy and an AD patients, respectively (Mandelkow and Mandelkow, 2011).

Moreover, *in vitro* and *in vivo* studies have demonstrated a connection between bacterial endotoxins and the development of AD. *Bacteroidetes*, which have been shown to increase in both CD and AD patients, are undoubtedly implicated. It has been proven that LPS from Gram-negative bacteria enhances amyloid fibrillogenesis, facilitates the deposition of amyloid in larger quantities, and promotes the formation of Tau (Kitazawa et al., 2005; Kahn et al., 2012; Asti and Gioglio, 2014). The direct involvement of Gram-negative bacteria, and the LPS and curli fimbriae they produce, in the pathomechanism of AD was proven by the study of Zhan et al. These authors provided unequivocal evidence for the co-localization of *E. coli*, and various compounds produced by them, including the curli fibers with amyloid plaques in postmortem brain tissue obtained from a patient with AD (Zhan, 2017). The CD-Aβ-AD association has been further confirmed by studies conducted by Sun et al. These authors demonstrated that after injection into the stomach wall of mice, Aβ1-42 oligomers were detected in the small intestine, vagus nerve, and brain after one year. Therefore, amyloid induced changes in the functioning of the gastrointestinal organs, ultimately contributing to amyloidosis in the central nervous system and AD-like dementia (Sun et al., 2020). These studies strongly suggest that Aβ oligomers from the gastrointestinal tract may cross into the brain, thereby participating in the pathogenesis of neurodegenerative diseases.

Cognitive impairment following retrograde transport of intra-GI administration of Aβ oligomers were demonstrated *in vivo* studies (Homolak et al., 2023).

**Figure 5:**
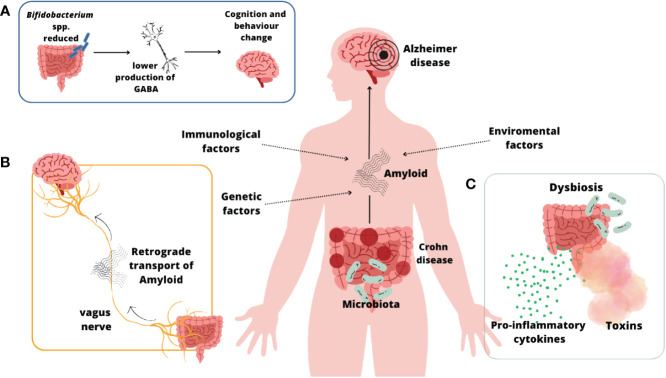
CD and AD connection (Mandelkow and Mandelkow, 2011; Villar-Piqué et al., 2016; Friedland and Chapman, 2017; Mehra et al., 2019; Li and Wen, 2022; Zeng et al., 2022; Xing et al., 2023)]**(A)**; The link between reduction in the quantity of *Bifidobacterium* spp., neurotransmitter GABA and developing of AD symptoms (Li and Wen, 2022); **(B)** Retrograde transport of amyloid fibers through vagus nerve (Zeng et al., 2022; Xing et al., 2023); **(C)** Leaky gut syndrome (Villar-Piqué et al., 2016; Mehra et al., 2019).

**Figure 5** shows some of mechanism mentioned above that take part in AD development on the base of IBD – CD.

Moreover, genetic and environmental risk factors, as described in the introduction of this Review, may contribute not only to the development of CD, but also to neurodegeneration. However, this is only speculation, as genetic meta-analyses conducted to date have not shown a link between AD and CD (Li and Wen, 2022; Zeng et al., 2022; Xing et al., 2023; Liao et al., 2024).

## 6 Conclusions

Based on the above evidence, AD can be added to the growing list of gastrointestinal microbiological diseases associated with disruptions in their microbiota. Given the constantly increasing amount of evidence, both from experimental and clinical studies, we now know that CD increases the risk of AD, and Aβ seems to be one of the links between these pathological conditions. The question that requires an answer and further research is whether the increased risk of developing AD in the course of CD is an implication or rather a co-occurrence, the cause of which lies somewhere deeper.

Although research on amyloid proteins produced by representatives of the microbiota and their impact on health and disease is still in the ‘crawl’ stage and requires significant time and scientific solutions. However, this topic is worth attention as possible interference with the microbiota or the products produced by it (including Aβ) could prove to be an effective therapeutic solution in the fight against neurodegenerative diseases, which effectively seem to have taken over humanity. One such direction is therapies that inhibit Aβ accumulation to prevent increased risk of AD due to colitis. In addition, therapeutic options aimed at inhibiting neutrophil infiltration offer great hope for the future. Also promising are studies of fecal calprotectin levels and Th1- and Th17-related cytokines in serum. Appropriate early detection of these biomarkers would allow the determination of CD disease activity and the implementation of effective treatment, thereby preventing complications, including the development of AD. Based on the studies described above, a future strategy for combating NDs seems to be the transplantation of synthetic intestinal microbiota. This concept would focus on producing such a preparation that would be enriched with probiotics beneficial to NDs patients. This method would be a more effective alternative to FMT in this group of patients.

## Author contributions

AD: Conceptualization, Data curation, Investigation, Resources, Supervision, Visualization, Writing – original draft, Writing – review & editing. JS: Investigation, Resources, Visualization, Writing – original draft. NS: Investigation, Resources, Software, Visualization, Writing – original draft. AM: Resources, Visualization, Writing – original draft. MS: Data curation, Investigation, Resources, Supervision, Visualization, Writing – original draft, Writing – review & editing.

## Glossary

**Table:**

AApoAl	apolipoprotein Al-derived amyloidosis
Aβ	β-amyloid
AD	Alzheimer’s disease
ALS	amyotrophic lateral sclerosis
ANP	atrial natriuretic peptide
APOE	apolipoprotein E
APP	amyloid precursor protein
ATG	autophagy related gene
ATTR-CM	transthyretin amyloid cardiomyopathy
B2M	β2-microglobulin
BBB	blood-brain barrier
CAG	cytosine–adenine–guanine
CARD	caspase activating recruitment domain
CCR6	chemokine receptor 6
CD	Crohn’s disease
chILD	children’s interstitial lung disease
CJD	Creutzfeldt-Jakob disease
CNS	central nervous system
CX3CR1^high^	intestinal CX3C chemokine receptor (high)
DLG5	discs large MAGUK (membrane associated guanylate kinases) scaffold protein 5
DSS	dextran sodium sulfate
EOAD	early-onset Alzheimer’s disease
FFI	fatal familial insomnia
FMT	fecal microbiota transplantation
Foxp3	forkhead box protein 3
FTD	fronto-temporal dementia
GABA	gamma-aminobutyric acid
GBA	gut-brain axis
GMBA	gut-microbiota-brain axis
GSD	Gerstmann-Straussler disease
HCCAA	hereditary cystatin C amyloid angiopathy
HD	Huntington's disease
HDL	high-density lipoprotein
HDL1	Huntington disease-like type 1
HIV	human immunodeficiency virus
HR	hazard ratio
IAPP	islet amyloid polypeptide (amylin)
IBD	inflammatory bowel disease
IBD5	inflammatory bowel disease 5
IL	interleukin
IL23R	interleukin-23 receptor
IRGM	immunity related GTPase M
JAK2	Janus kinase 2
LFS	Li-Fraumeni syndrome
LOAD	late-onset Alzheimer’s disease
LPS	lipopolysaccharide
LRRK2	leucine-rich repeat kinase 2
miRNA	microRNA
MMP	metalloproteins
mtDNA	mitochondrial DNA
ND	neurodegenerative disorders
NK	natural killer
NFkB	nuclear factor kappa light chain enhancer of activated B cells
NOD	nucleotide-binding oligomerisation domain
OCTN	organic cation transporters novel
OTA	ostertag-type amyloidosis
p53	regulatory protein important protein in the cell cycle, DNA repair and apoptosis initiation, mutated in human cancers
PCOS	polycystic ovary syndrome
PD	Parkinson’s disease
PiB-PET	11C-Pittsburgh compound B positron emission tomography
PMEL	pre-melanosomal protein
proSP-C	prosurfactant protein C
PrP	prion protein
PSEN	presenilin
RCC	renal cell carcinoma
ROR	retinoic acid related orphan receptor
RORγt	retinoic acid related orphan receptor γt
ROS	reactive oxygen species
RR	risk ratio
S100A9	S100 calcium-binding protein A9
SCFA	short-chain fatty acid
STAT3	signal transducer and activator of transcription 3
Tau	Tau protein
TDP-43	TAR DNA binding protein 43
Th	T helper
TNF-α	tumor necrosis factor alpha
TNFSF15	TNF superfamily member 15
TLR	toll-like receptor
Treg	regulatory T cells
TREM	triggering receptor expressed in myeloid/microglial cells
UC	ulcerative colitis
5hmC	5-hydroxymethyl cytosine
5mC	5-methyl cytosine.
